# MICROBIOME-KIDNEY METABOLISM PROVIDES INSIGHT INTO THE MECHANISM OF ICARIIN ALLEVIATES AGING IN OLDER FEMALE MICE

**DOI:** 10.1093/geroni/igae098.2840

**Published:** 2024-12-31

**Authors:** Xiaoang Li, Yiming Zhang, Liping Duan

**Affiliations:** Peking University Third Hospital, Beijing, Beijing, China (People’s Republic); Peking University Third Hospital, Beijing, Beijing, China (People’s Republic); Peking University Third Hospital, Beijing, Beijing, China (People’s Republic)

## Abstract

The average life expectancy of women in most countries and regions is significantly higher than men’s. Whether in rich developed countries or relatively poor third-world countries, women are more likely to live. Our previous study revealed that Icariin enhances youth-like features by attenuating the declined gut microbiota in older male mice. To further study the mechanism of icariin on delaying aging in older female mice on gut microbiota and metabolism, two age groups of C57BL/6J female mice (8 week- and 24 month- mice) were given a single dose of icariin at 100mg/kg by gavage for 15 days. The 16S rRNA gene pyrosequencing analyzed the fecal microbiome. We observed clear differences in gut microbial profiles between the young and older female mice. Interestingly, while older male mice did not show this effect, treatment with icariin increased the abundance of the short-chain fatty acid (SCFA)-producing bacteria Akkermansia muciniphila and Eubacterium ramulus in older female mice, bringing their levels close to those of untreated young female mice at the species level. Further, we used non-targeted metabolomics technology to detect all metabolites in the kidney. We found that the level of D-serine in the kidneys of older female mice was significantly lower than that of young female mice, and the metabolite levels were significantly increased after icariin treatment. SCFA and D-serine are gut microbial-derived products with kidney-protective properties. This study provides valuable insights into the molecular mechanisms of icariin, highlighting target characteristics and key pathways for slowing the aging process.

